# General practitioner practice-based pharmacist input to medicines optimisation in the UK: pragmatic, multicenter, randomised, controlled trial

**DOI:** 10.1186/s40545-020-00279-3

**Published:** 2021-01-04

**Authors:** Nadia Farhanah Syafhan, Sayer Al Azzam, Steven D. Williams, Wendy Wilson, Jayne Brady, Peter Lawrence, Mark McCrudden, Mustafa Ahmed, Michael G. Scott, Glenda Fleming, Anita Hogg, Claire Scullin, Robert Horne, Harblas Ahir, James C. McElnay

**Affiliations:** 1 Clinical and Practice Research Group, School of Pharmacy, Queen’s University Belfast, Belfast, BT9 7BL UK; 2 Department of Clinical Pharmacy, Faculty of Pharmacy, Universitas Indonesia, Depok, Indonesia; 3 Department of Clinical Pharmacy, Jordan University of Science and Technology, Irbid, Jordan; 4 Westbourne Medical Centre, Dorset, UK; 5 The Adam Practice, Dorset, UK; 6 Wingate Medical Centre, Liverpool, UK; 7 Pendle View Medical Centre, Nelson, UK; 8 Antrim Area Hospital, Antrim, UK; 9 Fern House Surgery, Essex, UK; 10 Douglas Grove Surgery, Essex, UK; 11 Medicines Optimisation Innovation Centre (MOIC), Antrim, UK; 12 School of Pharmacy, University College London, London, UK; 13 Merck Sharp & Dohme Limited, Hoddesdon, UK

**Keywords:** Clinical pharmacist, General practice, Practice-based pharmacist, Healthcare resource utilisation, Medicines optimisation

## Abstract

**Background:**

Changing demographics across the UK has led to general practitioners (GPs) managing increasing numbers of older patients with multi-morbidity and resultant polypharmacy. Through government led initiatives within the National Health Service, an increasing number of GP practices employ pharmacist support. The purpose of this study is to evaluate the impact of a medicines optimisation intervention, delivered by GP practice-based pharmacists, to patients at risk of medication-related problems (MRPs), on patient outcomes and healthcare costs.

**Methods:**

A multi-centre, randomised (normal care or pharmacist supplemented care) study in four regions of the UK, involving patients (*n* = 356) from eight GP practices, with a 6-month follow-up period. Participants were adult patients who were at risk of MRPs.

**Results:**

Median number of MRPs per intervention patient were reduced at the third assessment, i.e. 3 to 0.5 (*p* < 0.001) in patients who received the full intervention schedule. Medication Appropriateness Index (MAI) scores were reduced (medications more appropriate) for the intervention group, but not for control group patients (8 [4–13] to 5 [0–11] vs 8 [3–13] to 7 [3–12], respectively; *p* = 0.001). Using the intention-to-treat (ITT) approach, the number of telephone consultations in intervention group patients was reduced and different from the control group (1 [0–3] to 1 [0–2] vs 1 [0–2] to 1 [0–3], *p* = 0.020). No significant differences between groups were, however, found in unplanned hospital admissions, length of hospital stay, number of A&E attendances or outpatient visits. The mean overall healthcare cost per intervention patient fell from £1041.7 ± 1446.7 to £859.1 ± 1235.2 (*p* = 0.032). Cost utility analysis showed an incremental cost per patient of − £229.0 (95% CI − 594.6, 128.2) and a mean QALY gained of 0.024 (95% CI − 0.021 to 0.065), i.e. indicative of a health status gain at a reduced cost (2016/2017).

**Conclusion:**

The pharmacist service was effective in reducing MRPs, inappropriateness of medications and telephone consultations in general practice in a cost-effective manner.

*Trial registration:* ClinicalTrials.Gov, NCT03241498. Registered 7 August 2017—Retrospectively registered, https://clinicaltrials.gov/ct2/show/NCT03241498

## Background

As the population ages, the disease burden and number of comorbidities increases in individual patients [1, 2]. Medication-related problems (MRPs) are increasingly likely to arise in older patients since their medication is often more complex [3]. A medication-related problem (MRP) is defined as “an event or circumstance involving medication therapy that actually or potentially interferes with an optimum outcome for a specific patient” [4]. These problems have been found to lead to an increased incidence of hospital admissions, primary care physician and emergency department visits, and thus increase the cost of healthcare provision [5–7].

According to their report to the Policy Research Unit in Economic Evaluation of Health and Care Interventions, Elliot and colleagues (2018) estimated that primary care adverse drug reaction (ADR)-related hospital admissions in the UK have an annual cost of £83.7 million and cause over 627 deaths per annum [8]. Older age, previous ADR-related hospital admission and polypharmacy have been associated with a high prevalence of ADR-related hospital admissions [9, 10]. In addition, significant medication wastage has been found within general practice settings in England with an estimated £300 million worth of prescribed medications being wasted each year in primary and community care [11]. Optimisation of drug therapy and prevention of MRPs therefore has the potential to reduce health care expenditure, increase patient quality of life and save lives [12–15].

Medicines optimisation is defined as “a person-centred approach to safe and effective medicines use, to ensure people obtain the best possible outcomes from their medicines” [16]. One important aspect of the practice of evidence-based medicine is shared-decision making, an approach which involves seeking and sharing the best available evidence as guidance to decision making in individual patient care, while considering individual patient needs, preferences and values [17–19].

Greater engagement with patients and enhanced professional collaboration within health and social care settings all contribute to the medicines optimisation process. To support the medicines optimisation agenda a guide on medicines optimisation has been produced by The Royal Pharmaceutical Society, the aim of which is to ensure patients get the most benefit from their medicines [20].

Primary care systems internationally are increasingly starting to utilise a team-based approach to care delivery, with pharmacists increasingly recognised as a part of healthcare professional teams within primary care settings. This integration of pharmacy services into primary health care systems has been shown to have significant benefits including reduction in medication errors, effective identification and resolution of MRPs, improvements in medication adherence, improved patient outcomes, relief of work pressure on GPs, improved communication and co-operation between health professionals, and strengthened team working within primary care [21–25].

Systematic reviews have identified a variety of beneficial interventions and positive impacts when pharmacists and GPs work together. As the expansion of the role of pharmacists in the primary health care setting moves forward, more robust evidence on healthcare resource utilisation and cost-effectiveness, linked to well defined outcome measures, is required to determine whether practice-based pharmaceutical interventions are indeed effective, efficient and sustainable [26–30].

## Methods

The aim of the present research was to assess the impact of a medicines optimisation intervention, delivered in GP practices by practice-based pharmacists to patients at risk of MRPs, on patient outcomes and healthcare resource utilisation, i.e. the number of unplanned hospital admissions, A&E (accident and emergency) attendances, general practice consultations, outpatient visits and overall costs associated with health care delivery (including a cost utility analysis). Additional objectives were to assess the impact of the intervention on medicines optimisation measures (MRPs and medication appropriateness), self-reported medication adherence and humanistic outcomes (patient beliefs about medicines, health-related quality of life and patient satisfaction with the GP practice-based pharmacist service).

A pragmatic, prospective, multi-centre, randomised, controlled intervention study was conducted simultaneously in four different regions of the United Kingdom (UK) with two GP practices participating in the research in each of the four geographical areas, i.e. Northern Health and Social Care Trust (NHSCT), Northern Ireland; North West Coast Academic Health Science Network (AHSN), England; Wessex AHSN, England; and Eastern AHSN, England. In the UK GP practice populations are stable, i.e. a patient is registered with and attends a single GP practice where National Health Service provision is available at no cost to the patient.

The study was approved by the Office of Research Ethics Committees in Northern Ireland (ORECNI; 16/NI/0135). Research Governance approval was obtained from the Northern Health and Social Care Trust (NHSCT; NT16-0527-08) and the Health Research Authority NHS England (IRAS: 209697). ClinicalTrials.Gov registration no: NCT03241498.

Patient recruitment commenced on 28 November 2016 and was completed on 4 July 2017. The follow-up period was 6 months. A stratified approach to recruitment was used and patients were recruited sequentially, according to risk stratification, i.e. Stratum 1: adult patients aged 18 years old or over who have had at least one unplanned hospital admission or two or more A&E attendances in the previous 12 months and prescribed at least 6 regular oral or inhaled, long-term medicines; and Stratum 2: adult patients aged 18 years old or over who were prescribed at least 10 regular oral or inhaled, long-term medicines. The basis for this approach was to give priority to the recruitment of patients who were at higher risk of MRPs (stratum 1) and then move to recruiting patients in stratum 2 to reach intended patient numbers. Patients were excluded in the following cases: residing in a nursing home or a care home, considered unable to give written informed consent (e.g. Alzheimer’s disease), receiving palliative care, having had 4 or more unplanned admissions to hospital in the previous 6 months or participating in another research project or novel intervention scheme within the participating practice. The service, in the present study, was delivered by six GP practice-based pharmacists who operated as part of the wider primary care team within their respective GP practices. Each pharmacist received 2 days of intensive specialist training on medicines optimisation (including training on motivational interviewing) prior to commencing the research project.

The target, pragmatic sample size at each site was 50 intervention patients and 50 control patients, i.e. a total of 800 patients (400 intervention and 400 control patients) across the eight participating GP practices. The list of patients in each practice who met the inclusion criteria of stratum 1, followed by stratum 2 and who had no exclusion criteria was arranged in a random order (random.org) and then randomly assigned to control and intervention groups.

**Fig. 1:**
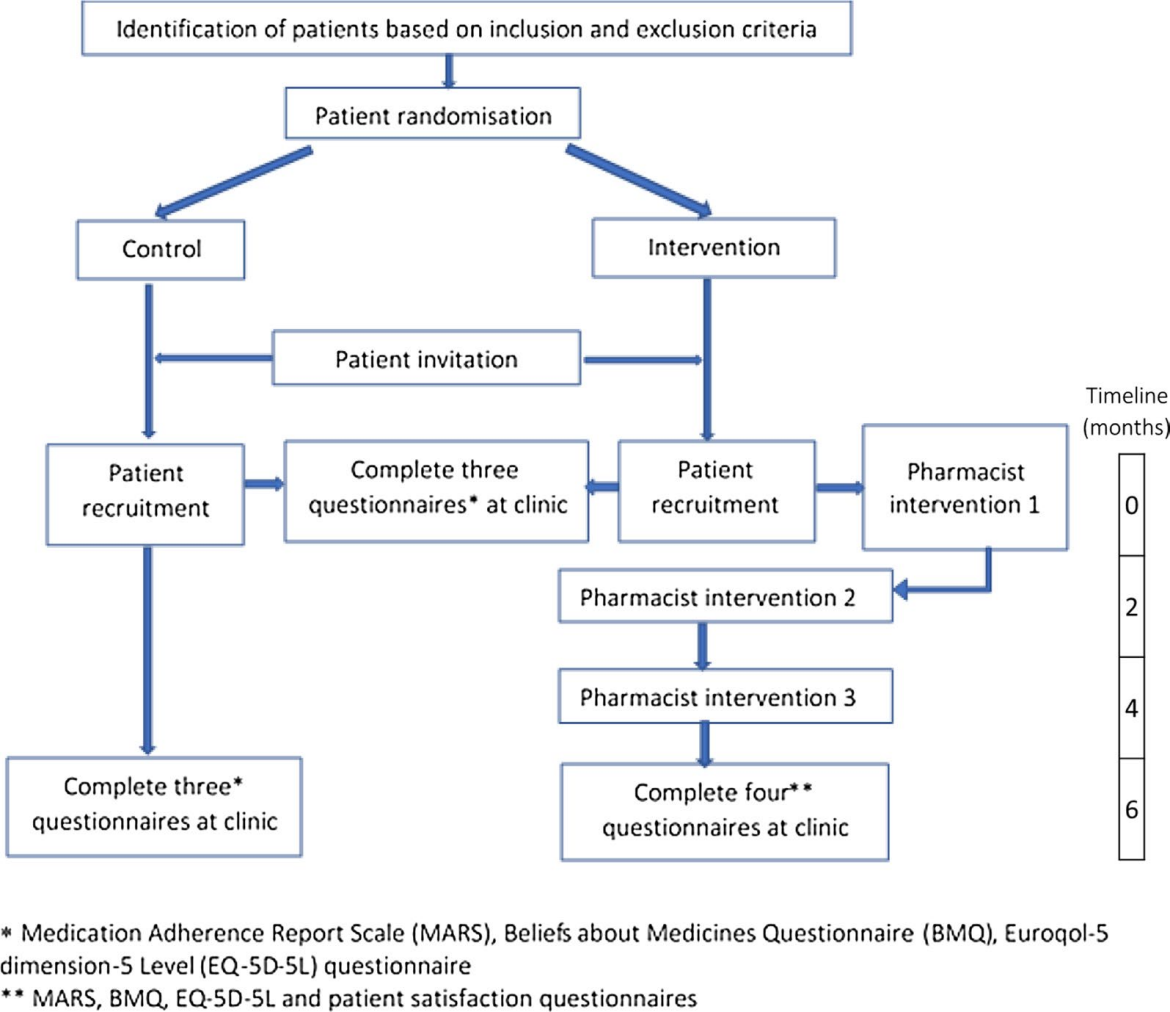
Study flowchart

Individual patients were sequentially invited to participate in the study via a letter of invitation (patient information sheet included) with telephone follow-up. A flyer, made available in GP practice waiting areas, was also used to help increase the recruitment rates. The invitation letter invited patients, who were willing to take part in the research, to arrange an appointment with the clinical pharmacist at the GP practice. Patients who attended an appointment and provided written, informed consent were assigned to the control or intervention arm of the study. The recruitment period was approximately 6 months in all practices. The study flowchart is presented in Fig. 1. In delivering the pharmacist interventions, the perceptions and practicalities theoretical framework [31] was applied, as recommended by the National Institute for Health and Care Excellence [32].

Prior to the first medicines optimisation intervention by the clinical pharmacist, he/she reviewed the patient’s medical notes, electronic record and laboratory data to identify potential medication-related issues. When the patient attended the practice, the pharmacist, through discussion with the patient, compiled a complete medication history (including non-prescription medicine use), reviewed medication adherence and appropriateness of medicines and discussed medication management with each patient. A pharmacist intervention guide, containing forms to complete for each patient at different stages of the research was available to assist in ensuring uniformity of the process within and across practices.

Having completed this process, the pharmacist created a list of potential MRPs in order to develop an individualised medicines optimisation intervention plan for each patient. The clinical pharmacist aimed at delivering a holistic, patient-centred service to each participating patient based on individual needs, expectations and outcomes that mattered to the patient. The interventions included recommending/making changes to medication regimens (in collaboration with GPs), personalised education and counselling on medication management, the correct use of medication administration devices and lifestyle factors, as appropriate for each patient. The pharmacist also, in discussion with the patient, drew up a list of treatment goals. Where necessary, the pharmacist referred the patient to another healthcare professional within the practice for management of other patient matters identified during the appointment, e.g. referred to a diabetic specialist nurse.

Having completed the intervention, the pharmacist produced a short report for the patient’s GP outlining actions taken and any further recommendations which required input from the GP. The same procedure was followed at subsequent patient visits at 2 and 4 months, building upon patient progress towards agreed goals. The control group were the normal care group. A bi-weekly teleconference involving all participating pharmacists and a site visit to each GP practice site were conducted by the university-based members of the research team as an integral part of project management to ensure that all the pharmacists were delivering a uniform service and completing all paperwork appropriately.

### Study outcome measures

The number of unplanned hospital admissions, A&E attendances, outpatient visits and general practice consultations in the 6 months prior to the commencement of the intervention and during the 6-month study period for each participating patient were obtained from the electronic record system (EMIS Web, SystmOne, or NIECR) at each participating GP practice. A cost utility analysis (CUA) [33] was then performed, with a NHS perspective adopted for the analysis. Data on resource utilisation together with intervention costs were used. Normalised unit costs were applied from national sources as detailed in Additional file 1. Other outcome measures used were number of MRPs, Medication Appropriateness Index (MAI) score, medication adherence report scale (MARS), beliefs about medicines questionnaire (BMQ) and Health-related quality of life (EQ-5D-5L) and patient satisfaction with the GP practice-based pharmacist service (bespoke questionnaire) (Additional file 2).

Random sample (random.org) of one-third of the total sample in every practice was identified for MAI scoring. A focus group discussion was performed at the end of the study to collect data on the views of the general practice-based pharmacists who delivered the medicines optimisation intervention, to help inform future study design. It will be explored in a separated paper.

### Data analysis

Quantitative data collected for participating patients were transferred to SPSS (version 25, USA inc) or STATA (version 15, StataCorp) for statistical analysis. Standard statistical methodology was used to assess the impact of clinical pharmacist interventions by comparing data from the intervention and control groups (including before and after analyses) using appropriate parametric or non-parametric tests. Standard cost utility methodological approaches [33, 34] were used to assess the economic impact of the interventions. Data on resource utilisation together with intervention costs were used. Responses to the EQ-5D were used to calculate QALYs (Quality Adjusted Life Years) gained over the follow-up period. The QALY value was determined by multiplying the utility value related to a given health state by the years observed in that state. The formula used to calculate QALYs was as follows [35]: QALYs gained = QALYs with intervention – QALYs without intervention.

Intention-to-treat analysis (ITT) was conducted for all patients who were randomised and recruited, i.e. including patients who did not complete the end of study questionnaires. Per protocol (PP) analysis was also conducted for patients who completed baseline and end of study questionnaires and received at least one pharmacist intervention.

## Results

**Fig. 2:**
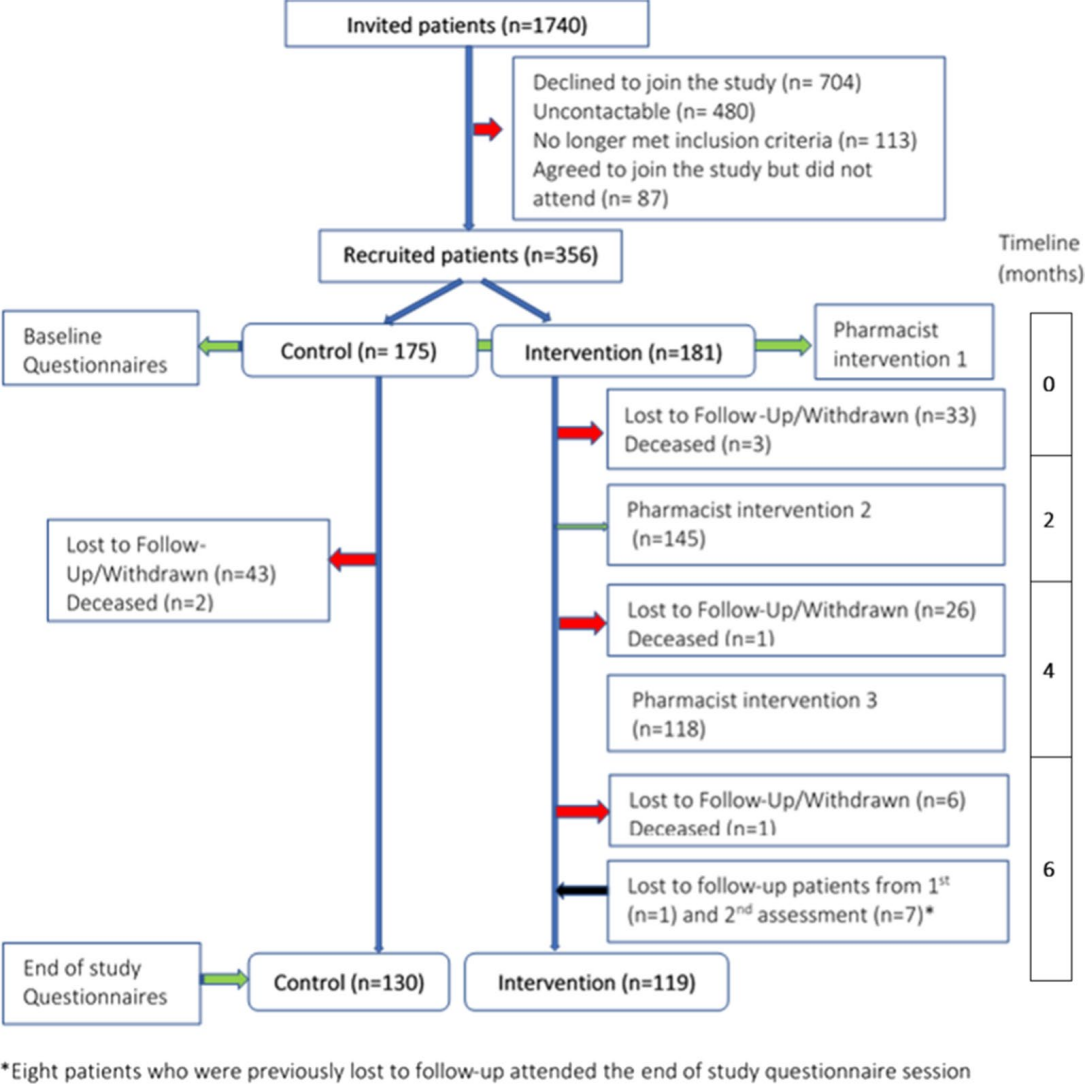
Flowchart of patient flows from invited to participate to completion of end of study questionnaires

A total of 1740 patients were invited (by letter) to participate in the study and 356 patients were recruited. Patient recruitment flows are presented in Fig. 2. The total number of patients recruited was lower than expected. Patient reasons for declining to participate included: not interested in participating in research project, did not have time to take part, e.g. carer for family member, happy with GP only service, did not want medications changed, already attended hospital pharmacist and selected for the control group. A number of patients were found to be unsuitable for participation i.e. were housebound, were acutely unwell, had moved out of the area, were unable to communicate or were admitted to hospital at the time of invitation to participate. The difference in number of deaths between groups was not statistically significant (P > 0.05).

### Patient characteristics

Characteristics of the 356 patients recruited are included in Table 1. Patients recruited mostly came from stratum 1 (64.3%). The average numbers of active medical problems and prescribed long-term medicines were approximately 7 and 10, respectively, in the control and intervention groups. The data (Table 1) indicate that patients were well matched between the control and intervention groups. The number of active medical problems was, however, slightly lower in the intervention group (7.3 ± 3.3 vs 7.9 ± 3.6; *p* < 0.05).

**Table 1:** Patient characteristics at baseline

Variable	Total	Control	Intervention	*p* value
	*N* = 356	*n* = 175 (49.2%)	*n* = 181 (50.8%)	
Stratum				0.752^a^
1 [*n* (%)]	229 (64.3)	114 (65.1)	115 (63.5)	
2 [*n* (%)]	127 (35.7)	61 (34.9)	66 (36.5)	
Age: [mean years ± SD]	67.9 ± 13.1	67.5 ± 12.6	68.5 ± 13.5	0.275^b^
Median [IQR]	70 [60.0–78.0]	69 [60.0–77.0]	71 [61.0–78.5]	
18–65 years [*n* (%)]	126 (35.4)	63 (36.0)	63 (34.8)	
> 65 years [*n* (%)]	230 (64.6)	112 (64.0)	118 (65.2)	
Range	25–96	26–94	25–96	
Sex				0.442^a^
Female [*n* (%)]	192 (53.9)	98 (56.0)	94 (51.9)	
Male [*n* (%)]	164 (46.1)	77 (44.0)	87 (48.1)	
Number of active medical problems: [mean ± SD]	7.6 ± 3.4	7.9 ± 3.6	7.3 ± 3.3	0.043^b^
Median [IQR]	7 [5–10]	8 [5–10]	6 [5–9]	
Number of repeat medicines: [mean ± SD]	10.3 ± 3.6	10.3 ± 3.5	10.3 ± 3.7	0.787^b^
Median [IQR]	10 [8–12]	10 [8–12]	10 [8–12]	

*SD* standard deviation, *IQR* interquartile range

^a^Chi-square test

^b^Mann–Whitney *U* test

### Medication-related problems

**Fig. 3:**
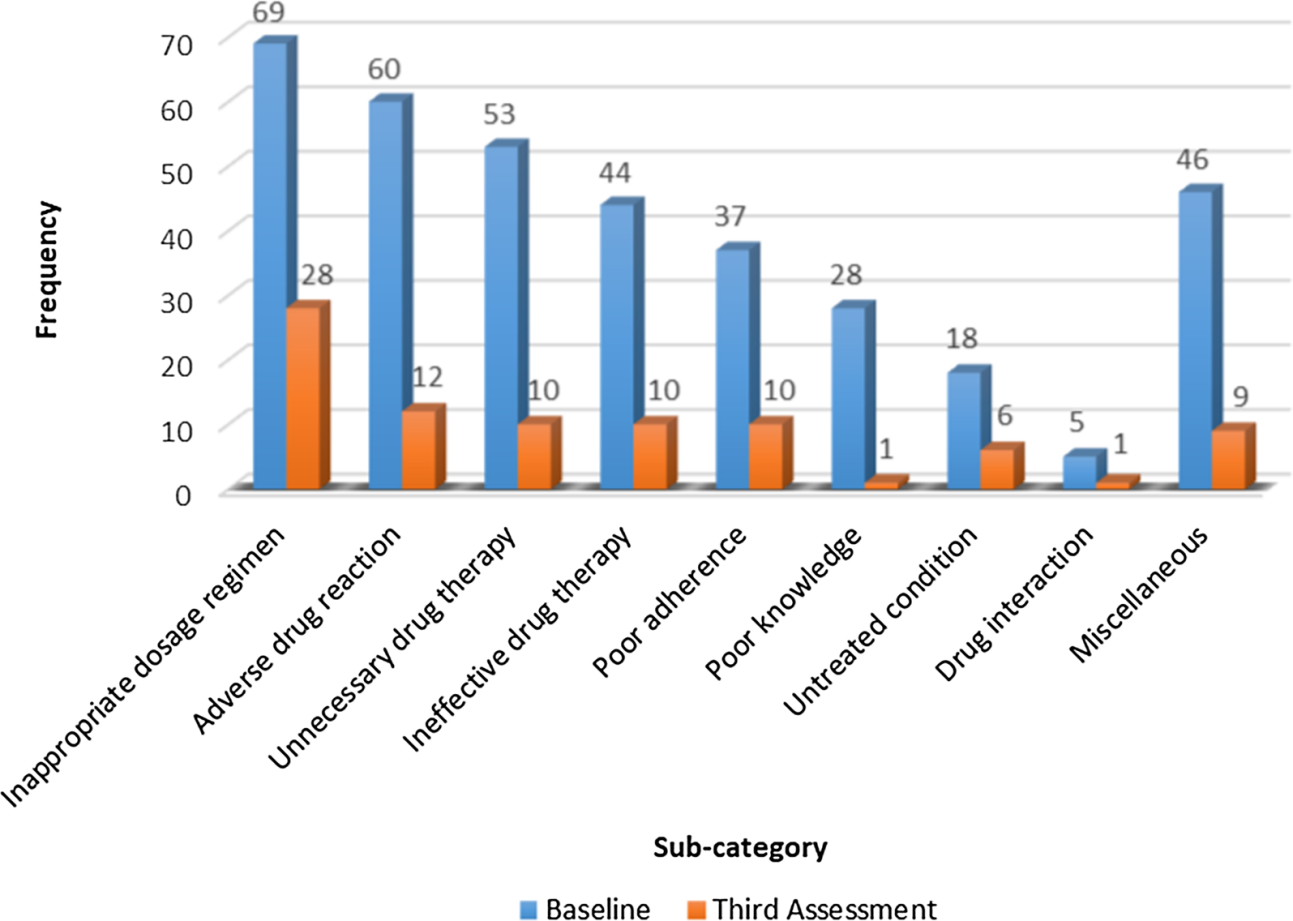
Sub-categories of medication-related problems identified at baseline and third assessment in patients (*n* = 118) who received three pharmacist interventions

The presence of identifiable MRPs provided the pharmacist with scope to improve medicine use (medicine optimisation). Analysis (baseline vs third assessment) of medication-related problem data was carried out in intervention patients who received three pharmacist interventions (*n* = 118). There was a significant decrease (*p* < 0.001) in median [IQR] numbers of MRPs per patient between baseline and the third assessment from 3.0 [2–4] to 0.5 [0–1]. A total 360 MRPs were identified at baseline. The most common sub-categories of MRPs identified were inappropriate dosage regimen (*n* = 69) followed by adverse drug reaction (*n* = 60) and unnecessary drug therapy (*n* = 53). A total of 87 MRPs were identified at the 3rd assessment; the most common sub-categories of MRPs identified at this final assessment were inappropriate dosage regimen (*n* = 28) followed by adverse drug reaction (*n* = 12) (Fig. 3). Unnecessary drug therapy, ineffective drug therapy and poor adherence sub-categories were all identified on 10 occasions at the final assessment. Statins, proton pump inhibitors and opioid analgesics were the most common classes of medications which led to interventions by the practice pharmacists (Additional file 3). Further details on MRP sub-categories at baseline and third assessment according to the classification devised by AbuRuz et al*.* (2006) [36], are shown in Fig. 3.

### Medication appropriateness index (MAI)

**Fig. 4:**
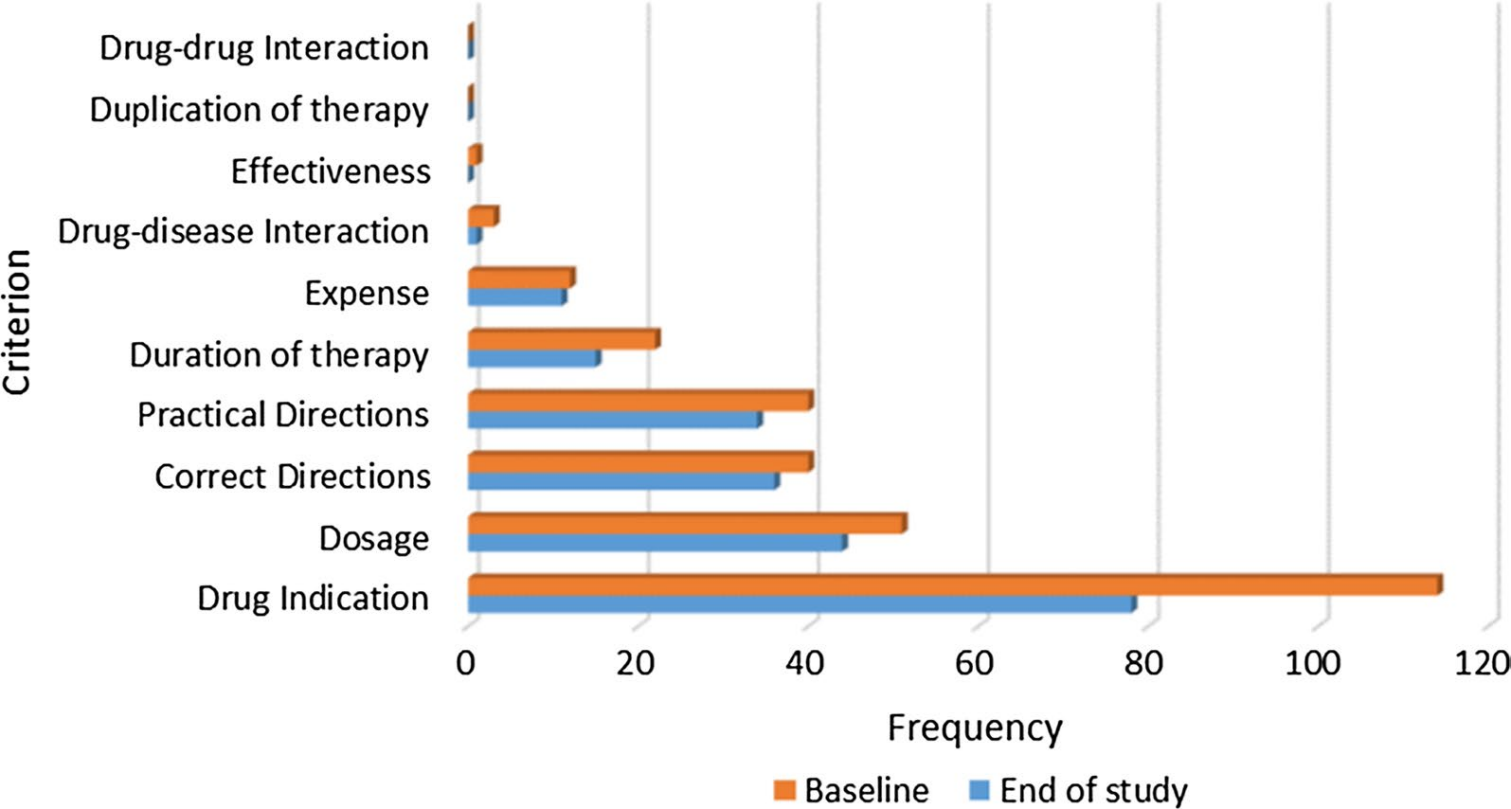
Frequency of inappropriate MAI criteria (*n* = 63 intervention patients; intention-to-treat group)

Due to the burden associated with assessment of medication appropriateness using the MAI tool, a random sample (random.org) of one-third of the total sample in every practice was identified for MAI scoring. Scoring was carried out for baseline and end of the study data, applying the ITT approach. Data indicated that there were significant improvements in medication appropriateness (decreases in MAI Score) in patients in the intervention group (Table 2). The most common criteria relating to inappropriateness in the intervention group at baseline and at the end of study related to drug indication followed by correct dosage and correct directions (Fig. 4). In interpreting the data in Fig. 4, attention should be given to the weighting of different criteria in the calculation of MAI scores, i.e. indication and effectiveness are triple weighted; dosage, correct directions, drug–drug interactions, or drug–disease interactions are double weighted; practical directions, duplication, duration of treatment and cost are single weighted.

**Table 2:** Medication appropriateness index (MAI) scores; ITT analysis

Outcome measures	Control			Intervention			*p* value
	*n* = 60 (48.8%)*			*n* = 63 (51.2%)*			
	Baseline	End	Difference^a^	Baseline	End	Difference^a^	
Number of medications scored (total)	620	601		628	602		
Mean ± SD^#^	10.3 ± 3.6	10.0 ± 3.9		10.0 ± 3.8	9.6 ± 3.9		
Median [IQR]^#^	10 [7.3–12.0]	10 [6.3–12.0]		9 [7.0–12.0]	9 [7.0–11.0]		
Summated MAI score per patient, mean ± SD	9.1 ± 7.3	9.1 ± 8.5	0.0 ± 4.0	9.6 ± 7.6	7.2 ± 8.1	2.4 ± 4.8	0.879^b^ ** < 0.001**^**c**^ **0.001**^d^
Median [IQR]	8 [313]	7 [312]	0 [0–0]	8 [4–13]	5 [0–11]	0 [0–5]	

Bold values denote statistical significance at the *p* < 0.05 level

*Random sample (random.org) of one-third of the total sample in every practice, ^#^Medications per patient

^a^Calculated as MAI score at baseline – MAI score at end of study

^b^Wilcoxon signed rank test in control group, between baseline and end of study

^c^Wilcoxon signed rank test in intervention group, between baseline and end of study

^d^Mann–Whitney *U* test between groups at end of study for MAI difference

### Questionnaire-based outcomes

Within this section, only the PP (patients who completed baseline and end of study questionnaires and received at least one pharmacist intervention) data were utilised.

### Medication adherence

The MARS scores at baseline and end of study indicated that patients, by self-report, were mostly adherent to their medication with median MARS scores of 24 throughout the study period. Based on clinical pharmacist feedback gathered through a focus group discussion conducted at the end of this study, participating patients were well-motivated and generally reported few problems with their medication. A typical pharmacist statement was as follows:

I think the real thing that was really obvious with the patients who actually came in, was that they are quite well-motivated…. Their medication appeared to fit and they were quite well.

### Beliefs about medicines

There were also no significant within group or between group differences in BMQ scores (baseline vs end of study assessment). Both control and intervention group patients had median necessity scores (21 and 20, respectively, throughout the study period) that were higher than median concern scores (13 and 13 at baseline; 15 and 13 at end of study). No statistically significant variation was noted over time, with the median necessity/concerns differential remaining constant between baseline and 6 months in both control (7.5) and intervention (7.0) patients.

### Health-related quality of life

Control group patients showed a slight decline from baseline in the EQ-5D-5L utility scores at the end of study (from a median of 0.616 to 0.596), while there was no change in this score in the intervention group patients (medians of 0.647 to 0.648). Both groups showed a decline in their visual analogue scale (VAS) general health state scores over the follow-up period; in the control group this decline was statistically significant (from median 60 to 50; *p* < 0.05) while the score was not statistically significant in the intervention group patients (from median 65 to 60; *p* > 0.05). There was no significant difference in the scores between control and intervention groups.

### Patient satisfaction questionnaire

Additional file 4 illustrates the results obtained from the bespoke patient satisfaction questionnaire. It is clear that at the end of the study follow-up period more than 75% of the intervention patients who responded to the satisfaction questionnaire (*n* = 101) were satisfied with all aspects of the service. The highest satisfaction levels of 92.1% and 91.1% were demonstrated in response to statements one and two, respectively, which related to overall view of the new service and on beneficial information given by the pharmacist.

### Healthcare resource utilisation

From the 356 patients recruited, 14 patients whose healthcare resource utilisation data were not accessible and seven deceased patients were excluded from the healthcare resource utilisation aspect of the research. A total of 335 patients were therefore considered (control plus intervention), i.e. ITT approach taken. Patients with a length of stay (LOS) of 20 days or greater were categorised as outliers and excluded from the analysis [37–39] as such a LOS was considered more likely to be disease-related [40, 41] rather than medication-related. Based on this latter exercise, 5 patients were excluded, i.e. data from a total of 330 patients were included in the analyses of healthcare resource utilisation. The number of unplanned hospital admissions, number of telephone consultations in general practice and numbers of total general practice consultations (both face-to-face and telephone) decreased significantly (*p* < 0.05) in the intervention group over the 6-month follow-up period when compared with the prior 6 months. A significant reduction was found only in the median number of telephone consultations over the 6-month follow-up period when compared with control group data (*p* < 0.05). A decrease in the number of unplanned hospital admissions, number of face-to-face consultations in general practice and numbers of total general practice consultations (both face-to-face and telephone) were also recorded in the control group, but these changes were not statistically significant (*p* > 0.05). No statistical differences were found in the median length of stay, median number of A&E attendances and median number of outpatient visits within and between groups, pre- and post-initiation of the new practice-pharmacist service (Table 3).

**Table 3:** Healthcare resource utilisation in control and intervention group patients at baseline and at 6-month follow-up

Outcome measures	Control			Intervention			*p* value
	Baseline (6 months pre-study)	6-month follow-up	Difference^#^	Baseline (6 months pre-study)	6-month follow-up	Difference^#^	
	*n* = 161* (48.8%)	*n* = 161* (48.8%)		*n* = 169* (51.2%)	*n* = 169* (51.2%)		
Number of unplanned hospital admissions (total)	46	33		40	21		
Mean ± SD	0.3 ± 0.67	0.2 ± 0.58	0.1 ± 0.8	0.2 ± 0.51	0.1 ± 0.40	0.1 ± 0.6	0.279^b^ **0.023**^**c**^ 0.501^d^
Median [IQR]	0 [0–0]	0 [0–0]	0 [0–0]	0 [0–0]	0 [0–0]	0 [0–0]	
Number of patients admitted (total)	34	24		34	17		
Length of stay (days) (total)	148	97	-	118	74	-	
Mean ± SD	4.4 ± 4.4	4.0 ± 3.9	-	3.5 ± 4.1	4.4 ± 3.8	-	0.733^e^
Median [IQR]	3.0 [1.0–7.0]	2.5 [1.0–6.5]	-	1.5 [1.0–4.3]	3.0 [1.0–7.5]	-	
Number of A&E attendances (total)	45	57		43	37		
Mean ± SD	0.3 ± 0.6	0.4 ± 0.8	-0.1 ± 0.9	0.3 ± 0.5	0.2 ± 0.4	0.0 ± 0.6	0.326^b^ 0.433^c^ 0.333^d^
Median [IQR]	0 [0–0]	0 [0–0]	0 [0–0]	0 [0–0]	0 [0–0]	0 [0–0]	
Number of face-to-face consultations in general practice^a^ (total)	827	741		791	722		
Mean ± SD	5.1 ± 4.2	4.6 ± 3.8	0.5 ± 3.9	4.7 ± 3.4	4.3 ± 3.4	0.4 ± 3.4	0.087^b^ 0.069^c^ 0.953^d^
Median [IQR]	4 [2–7]	4 [2–6]	1 [-2–1]	4 [2–7]	4 [2–6]	1 [-2–2.5]	
Number of telephone consultations in general practice^a^ (total)	300	300		321	239		
Mean ± SD	1.9 ± 3.1	1.9 ± 2.9	0.0 ± 2.6	1.9 ± 2.7	1.4 ± 2.0	0.5 ± 2.4	0.481^b^ **0.006**^**c**^ **0.020**^**d**^
Median [IQR]	1 [0–2]	1 [0–3]	0 [-1–1]	1 [0–3]	1 [0–2]	0 [0–1]	
Total (face and telephone) number of General Practice consultations^a^	1127	1041		1112	961		
Mean ± SD	7.0 ± 5.9	6.5 ± 5.3	0.5 ± 5.1	6.6 ± 4.9	5.7 ± 4.3	0.9 ± 4.4	0.284^b^ **0.007**^**c**^ 0.227^d^
Median [IQR]	6 [3–9]	5 [3–9]	0 [− 2–3]	6 [3–9]	5 [2–8]	1 [− 2–4]	
Number of outpatient visits (total)	453	453		487	430		
Mean ± SD	2.8 ± 2.9	2.8 ± 2.9	0.0 ± 2.3	2.9 ± 3.1	2.5 ± 3.1	0.3 ± 2.6	0.841^b^ 0.074^c^ 0.136^d^
Median [IQR]	2 [1–4]	2 [0–4]	0 [− 1–1]	2 [1–4]	2 [0–4]	0 [− 1–2]	

Bold values denote statistical significance at the *p* < 0.05 level

*From 356 patients, 14 patients’ healthcare resource utilisation data were not available, seven patients were deceased, five patients had 20 or more days of length of stay at baseline or 6-month follow-up

^#^Calculated as utilisation at baseline minus utilisation at end of study

^a^Consultation with general practitioner, pharmacist and nurse practitioner

^b^Wilcoxon signed rank test (within control group, between baseline and 6-month follow-up)

^c^Wilcoxon signed rank test (within intervention group, between baseline and 6-month follow-up)

^d^Mann–Whitney *U* test (between control vs intervention for the utilisation difference from baseline)

^e^Mann–Whitney *U* test (between control vs intervention at 6-month follow-up)

### Health economic outcomes

Using the same datasets as for healthcare resource utilisation (*n* = 330), the total cost of healthcare resource utilisation in intervention group patients, including intervention costs, showed a significant (*p* = 0.032) decrease when 6 months of pre-intervention data were compared with 6 months of post-intervention data. Using the ITT approach, the mean overall cost per patient fell from £1041.7 ± 1446.7 to £859.1 ± 1235.2 (Table 4). There were, however, no statistically significant differences (*p* > 0.05) in the cost of the individual cost elements across the dataset with the exception of costs associated with telephone consultations in general practice and total costs of all general practice consultations. Reductions in cost associated with number of telephone consultations in general practice was significantly different when compared with the control group (*p* < 0.05). A decrease in the overall cost per patient, cost of unplanned hospital admissions, face-to-face consultations in general practice and total general practice consultation were also recorded in the control group, but this decrease was not statistically significant (*p* > 0.05). No statistical differences were found in the cost of A&E attendances and outpatient visits within and between groups, pre- and post-initiation of the new GP practice-based pharmacist service.

**Table 4:** Cost of healthcare resource utilisation in control and intervention group patients at baseline and at 6-month follow-up

Outcome measures	Control			Intervention			*p* value
	Baseline (6 months pre-study)	6-month follow-up	Difference^#^	Baseline (6 months pre-study)	6-month follow-up	Difference^#^	
	*n* = 161* (48.8%)	*n* = 161* (48.8%)		*n* = 169* (51.2%)	*n* = 169* (51.2%)		
Cost of unplanned hospital admissions^a^ (total)	83,483	65,853		64,873	43,425		
Mean ± SD	518.5 ± 1438.0	409.0 ± 1446.6	109.5 ± 2027.7	383.9 ± 1224.1	257.0 ± 979.9	126.9 ± 1453.7	0.305^g^ 0.230^ h^ 0.743^i^
Median [IQR]	0 [0–0]	0 [0–0]	0 [0–0]	0 [0–0]	0 [0–0]	0 [0–0]	
Cost of A&E attendances^b^ (total)	6525	8265		6235	5365		
Mean ± SD	40.5 ± 86.1	51.3 ± 112.8	− 10.8 ± 127.2	36.9 ± 77.6	31.7 ± 64.2	5.2 ± 86.5	0.326^g^ 0.433^h^ 0.333^i^
Median [IQR]	0 [0–0]	0 [0–0]	0 [0–0]	0 [0–0]	0 [0–0]	0 [0–0]	
Cost of face-to-face consultations in General practice^c^ (total)	21,865	21,101		21,752	19,361		
Mean ± SD	135.8 ± 102.2	131.1 ± 106.2	4.8 ± 111.7	128.7 ± 101.2	114.6 ± 94.1	14.2 ± 103.4	0.511^g^ 0.081^h^ 0.467^i^
Median [IQR]	112 [66–186]	108 [56–182]	10 [− 56–56]	108 [46–200]	87 [36–174]	10 [− 40–72]	
Cost of telephone consultations in general practice^d^ (total)	4919.0	4916.0		5262.0	3564.5		
Mean ± SD	30.6 ± 52.4	30.5 ± 47.8	0.0 ± 43.6	31.1 ± 47.4	21.1 ± 32.0	10.0 ± 38.5	0.619^g^ ** < 0.001**^** h**^ **0.007**^i^
Median [IQR]	18.0 [0.0–36.0]	18.0 [0.0–39.8]	0.0 [− 18.0–16.8]	18.0 [0.0–52.5]	7.5 [0.0–28.0]	0.0 [0.0–19.5]	
Cost of total general practice consultation (total)	26,784.0	26,017.0		27,014.0	22,925.5		
Mean ± SD	166.4 ± 130.3	161.6 ± 131.1	4.8 ± 127.8	159.8 ± 125.8	135.7 ± 106	24.2 ± 115.7	0.551^g^ **0.014**^**h**^ 0.194^i^
Median [IQR]	138.0 [73.0–218.0]	133.0 [72.0–222.0]	10.0 [− 59.0–65.5]	136.0 [56.0–236.5]	107.5 [52.0–200.0]	15.0 [− 43.5–90.0]	
Cost of outpatient visits^e^ (total)	72,480	72,480		77,920	68,800		
Mean ± SD	450.2 ± 465.9	450.2 ± 467.2	0.0 ± 360.9	461.1 ± 496.8	407.1 ± 488.8	54.0 ± 418.57	0.934^g^ 0.074^h^ 0.136^i^
Median [IQR]	320 [160–640]	320 [0–640]	0 [− 160–160]	320 [160–640]	320 [0–640]	0 [− 160–320]	
Total healthcare resource utilisation cost^f^ (total)	189,272	172,615		176,042	145,184		
Mean ± SD	1175.6 ± 1619.3	1072.1 ± 1635.0	103.5 ± 2104.6	1041.7 ± 1446.7	859.1 ± 1235.2	182.6 ± 1579.2	0.276^g^ **0.032**^** h**^ 0.491^i^
Median [IQR]	552 [313.5–1251.0]	572 [208.0–1168.3]	21.0 [− 267.5–416.5]	618 [279.0–1124.0]	477 [232.3–927.3]	95.5 [− 241.8–472.0]	

Bold values denote statistical significance at the *p* < 0.05 level

*From 356 patients, 14 patients’ healthcare resource utilisation data were not available, seven patients were deceased, five patients had 20 or more days of length of stay at baseline or 6-month follow-up

^#^Calculated as utilisation at baseline minus utilisation at end of study

^a^Cost of non-elective inpatient stay (short stay) i.e. 1–2 days £608, 3–6 days £3079, non-elective excess bed day (long stay more than 6 days, attracted cost for every day over of £437)

^b^A&E attendances £145

^c^GP £36, Pharmacist £15, nurse practitioner £10

^d^GP £18, pharmacist £7.5, nurse practitioner £5

^e^Outpatient visit £160

^f^All healthcare resource utilisation cost including intervention cost. The 1st, 2nd, 3rd appointment costs were £15, £8.5 and £7.5

^g^Wilcoxon signed rank test (within control group, between baseline and 6-month follow-up),

^h^Wilcoxon signed rank test (within intervention group, between baseline and 6-month follow-up)

^i^Mann–Whitney *U* test (between control vs intervention for the cost difference from baseline).

### Cost utility analysis

The PP approach was used for the cost utility analysis due to the importance of having EQ-5D-5L data for the calculations. From the 249 patients in the PP group, five patients whose healthcare resource utilisation data were not accessible, eight patients who had missing end-point EQ-5D-5L utility value data and four patients with a LOS of 20 days or more were excluded, resulting in the inclusion of a total sample of 232 patients in the analysis. The overall intervention group costs for the 6-month follow-up period were lower than the control group costs (£810.1 ± 1133.9 vs £1039.1 ± 1562.8 per patient) while the EQ-5D-5L utility scores were marginally higher in the intervention group (0.554 ± 0.321 vs 0.506 ± 0.323). The mean incremental total cost was − £229.0 (95% CI − 594.6, 128.2) and a mean incremental QALY was 0.024 (95% CI − 0.021 to 0.065) are presented in Additional file 5. An ICER (incremental cost–effectiveness ratio) was not calculated because the medicines optimisation intervention delivered by practice pharmacists clearly demonstrated a dominant strategy [42]. The cost–effectiveness plane, after 1000 bootstrap replications, represents uncertainty around the cost and effects estimates (Additional file 6). Although the incremental costs and effects density straddled all four quadrants of the cost–effectiveness plane, the majority of the points lay in the dominant (south-east) quadrant, indicating improved outcomes linked with reduced cost.

The impact of increasing the total healthcare cost by 50%, increasing the pharmacist intervention cost by 50% and decreasing the QALY gained by 50% were explored during sensitivity analysis (Additional file 7). The results showed that pharmacist medicines optimisation intervention remained the dominant strategy when the intervention cost and QALY were varied. A cost saving was no longer recorded when the total healthcare cost was increased by 50%; however, the resulting ICER of £6700 per QALY was still very considerably lower than the £20,000 per QALY NICE threshold [43, 44].

## Discussion

The RCT study design used in the present study was considered, based on the pyramid of evidence [45], as the best way to gather new data on the impact of practice-based pharmacists on economic and humanistic outcomes due to its ability to reduce confounding factors and bias [45–47]. Due to a general lack of well-designed studies in the primary care setting, RCTs are particularly important for establishing an evidence base pertinent to clinical decisions and proposed new services [48]. A pragmatic approach to define the target sample size was used, however, if the predicted difference in overall healthcare costs was £250 per patient over 6 months [49], this sample size was needed to give 80% power to detect a cost difference at the 5% significance level with a possible 20% loss to follow-up.

The MRP analysis was carried out only for patients who had three pharmacist visits to help demonstrate the maximal achievable impact of the intervention. The median number of three MRPs per patient at baseline in the present study was comparable to key findings of a systematic review involving general practice patients in the United States, Sweden, England and Scotland. In the latter study, the patients had an average of 3.2 MRPs [50]. The present study indicated that the pharmacist intervention decreased the median number of MRPS per patient from a median of 3.0 to 0.5, i.e. a decrease of 2.5 per patient. This reduction in MRPs is higher than the results from another RCT which evaluated a pharmacist-led intervention in primary care in Sweden where the mean decrease was 0.43 (from 1.73 to 1.31) [51]. In a pre–post intervention study in Australia, which involved the integration of pharmacists into general practice clinics, a median decrease of MRPs from 2 to 0 was recorded. In this latter study, non-adherence, untreated indication and inappropriate drug were the most commonly identified MRP categories [24]. The difference could be explained by different populations in the studies, e.g. lower risk and fewer MRPs at baseline. It is clear therefore from the present study, and from previous research, that pharmacist input can have a beneficial impact on MRPs across a range of settings internationally [21, 23, 24, 29, 52]. Since identification, resolution or prevention of MRPs are key functions of pharmaceutical care delivered by a pharmacist, this finding is to be expected. Statins were the class of medications most frequently identified in causing MRPs in the present study. Interestingly a study carried out in Scotland noted that statin prescribing can be improved in high-risk patients in primary care via a pharmacist-led collaborative intervention [53].

Regarding medication appropriateness, the present study involved random samples of one-third of patients within each GP practice. The data clearly indicated that the pharmacist intervention improved medication appropriateness as measured by the decline of summated median scores. Although lower than some hospital-based studies [54–56], the improvements align with the findings from a Cochrane Review of studies which evaluated pharmaceutical care interventions in older people receiving polypharmacy [57, 58]. A number of studies have linked the resolution of MRPs and/or improvements in the medication appropriateness to a reduction of adverse drug-related events [59], improvement of clinical outcomes [60], and reduction of hospital admission, A&E attendances and total healthcare costs [61–63].

Self-reported adherence, as assessed by the MARS, was almost perfect across the whole study period for both intervention and control group patients. As also reported in focus group discussion, participating patients were well-motivated and therefore, there was no scope for pharmacists to significantly improve self-reported patient adherence.

The results of studies to date on the impact of pharmacist interventions in primary care settings are, however, variable. A number of studies including systematic reviews and meta-analysis have reported no significant impact of a range of pharmacist interventions in primary care on unplanned hospital admissions and length of stay [23, 24, 51, 64, 65], A&E attendances [23, 65], GP consultations [24, 51, 66] and outpatient visits [23, 66]. On the other hand, two studies have shown a positive impact on unplanned hospital admissions [49] and A&E attendances [66]. In the former study the mean number of unplanned hospital admissions significantly dropped over a 6-month follow-up period in the intervention group (from 0.09 ± 0.35 to 0.05 ± 0.23; *p* = 0.007) and increased in the control group (from 0.05 ± 0.25 to 0.07 ± 0.36; *p* = 0.106) [49]. A significantly lower rate in A&E attendances in intervention patients (compared with usual care) over a 3-year period (0.0 ± 0.2 vs 0.1 ± 0.4; *p* < 0.001) was demonstrated in the second study [66]. Although our study findings regarding the number of unplanned hospitalisation and length of stay in intervention groups over the 6-month follow-up period were not statistically different when compared with the control group, a significant decrease was found in the number of unplanned hospital admissions in the 6 months post vs the 6 months pre-intervention (median 0 [0–0] to 0 [0–0], mean 0.2 to 0.1; *p* < 0.05). A decrease was also noted in the control group, but this was a smaller reduction which was not statistically significant (median 0 [0–0] to 0 [0–0], mean 0.3 to 0.2; *p* > 0.05). The present study demonstrated significant reductions in the number of telephone consultations in general practice over the 6-month follow-up period when compared with the prior 6 months (from median 1 [0–3] to 1 [0–2], mean 1.9 to 1.4; *p* < 0.05) in intervention group and this was also significantly different from the control group (*p* < 0.05). In the UK, within the NHS, there are considerable work-load pressures on GPs and the reduction in telephone consultations noted in the present study could help reduce such pressures.

The health economic impact of pharmacist interventions in primary care have been reported in other studies in which such interventions have been shown to reduce costs (or at least, not add significantly to costs) while providing benefits over usual care [23, 49, 66, 67]. An overall cost saving of approximately £250 per patient has, for example, been reported in a study performed in Spain which evaluated medication review and follow-up services in older patients receiving polypharmacy [49]. In the present study the mean cost saving regarding healthcare resource utilisation was £213 per patient (£859.1 ± 1235.2 in intervention vs £1072.1 ± 1635.0 in control group patients). The large variability in costs and the relatively low sample size, however, meant that these cost reductions were not statistically significant. The results from the cost utility analysis identified the practice-based pharmacist intervention as the dominant strategy, i.e. positive impact on quality of life (mean incremental QALY of 0.024) and reduced overall costs (£229). These data are similar in form to those obtained in an outpatient clinic-based study that demonstrated a pharmacy-led management programme for Chronic Obstructive Pulmonary Disease (COPD) patients as the dominant strategy with a gain in QALYS of 0.065 and a cost saving of £671.59 per patient [68].

The present study had a number of limitations. Firstly, the sample size was smaller than planned; the number of patients lost to follow-up was also higher than anticipated, thus reducing the statistical power of the study. Face-to-face pharmacist follow-up at 2 and 4 months formed the intervention schedule in the present research. This intervention schedule was considered burdensome by some patients and may have influenced the lack of attendance at scheduled follow-up sessions and/or patient withdrawal. A further limitation was the likelihood that patients who were normally housebound did not join the study. Such patients are often multimorbid, receive polypharmacy and could potentially benefit more from medicines optimisation than their more mobile peers. The MRPs analysis was carried out only for patients who had three pharmacist visits. Deceased patients were excluded from the healthcare resource utilisation aspect of the research, and therefore the latter was not a pure ITT analysis. A relatively short time of follow-up (6 months after the first intervention) was another limitation of this study. It may take longer for the decrease in MRPs and improved medication appropriateness to have an impact on the healthcare utilisation outcome measures used, particularly unplanned hospital admissions. Finally, data on GP out-of-hours consultations were not captured as part of the study.

## Conclusion

It can be concluded that the practice-based pharmacist service in UK to adult patients who were at risk of MRPs was an effective intervention for reducing MRPs, inappropriateness of medications and the number of telephone consultations in general practice. There were positive trends in the data regarding the impact on healthcare utilisation outcomes, but in general these did not reach statistical significance (*p* < 0.05) within the 6-month follow-up period in intervention patients when compared with control patients. The results from the cost utility analysis suggest that this service is cost effective, and that improved health-related quality of life was achieved in intervention patients at an overall reduced cost.

## Supplementary Information

**Additional file 1.** Unit costs (£) used in healthcare resource utilisation calculations.

**Additional file 2.** Secondary outcome measurement details.

**Additional file 3.** Classes of medications attracting GP practice-based pharmacist interventions at baseline and the third assessment in intervention patients who received three pharmacist interventions.

**Additional file 4.** Patient satisfaction questionnaire results.

**Additional file 5.** Cost utility analysis using the mean data over the 6-month study period (per protocol approach).

**Additional file 6.** The cost–effectiveness plane.

**Additional file 7.** Sensitivity analysis associated with the cost utility analysis.

## Data Availability

The data used to support the findings of this study are included in this published article and its supplementary information files.
