# Supplementary material for: General practitioner practice-based pharmacist input to medicines optimisation in the UK: pragmatic, multicenter, randomised, controlled trial
Source: J Pharm Policy Pract. 2021 Jan 4;14:4. doi: 10.1186/s40545-020-00279-3 (PMC7784025; doi:10.1186/s40545-020-00279-3)
Supplement: Supplementary file 1 — Additional file 1. Unit costs (£) used in healthcare resource utilisation calculations. [file 40545_2020_279_MOESM1_ESM.docx]

**Additional file 1.** Unit costs (£) used in healthcare resource utilisation calculations

| **Service** | **Unit cost**  **(£, 2016)** | **Details** | **Source** |
| --- | --- | --- | --- |
| **Secondary care costs** | | | |
| Non-elective inpatient stay (short stay) | 608 | 1-2 days | Trust Financial Returns (TFR H, 2013/14), including inflation rate for 2016 [1] |
| Non-elective inpatient stay (long stay) | 3079 | 3-6 days |  |
| Non-elective excess bed day (long stay) | 437 | > 6 days |  |
| Accident and Emergency attendance | 145 | Per attendance |  |
| Outpatient visit | 160 | Per visit |  |
| **General Practice costs** | | | |
| General practitioner face-to face consultation | 36 | Per 10 minute consultation | Unit Costs of Health and Social Care (2016) [2] |
| General practitioner telephone consultation | 18 | Per 10 minute consultation |  |
| Pharmacist face-to face consultation | 15 | Per 10 minute consultation | Unit Costs of Health and Social Care (2014) [3] |
| Pharmacist telephone consultation | 7.5 | Per 10 minute consultation |  |
| Practice nurse face-to-face consultation | 10 | Per 10 minute consultation | Unit Costs of Health and Social Care (2016) [2] |
| Practice nurse telephone consultation | 5 | Per 10 minute consultation |  |
| **GP practice-based pharmacist input** |  |  |  |
| At first appointment | 15 | Per consultation | Chemist and Druggist (2017) [4] |
| At second appointment | 8.5 | Per consultation |  |
| At third appointment | 7.5 | Per consultation |  |
| TFR H: Trust Financial Returns Hospital, PSSRU: Personal Social Services Research Unit. | | | |

1. Trust Financial Returns, Health and Social Care, Department of Health, N. Ireland. 2013/2014.
2. Curtis L, Burns A. Unit costs of health and social care. Canterbury:
   Personal Social Services Research Unit (PSSRU), University of Kent. 2016. <https://www.pssru.ac.uk/pub/uc/uc2016/full.pdf> . Accessed 19 December 2017.
3. Curtis L. Unit costs of health and social care. Canterbury:
   Personal Social Services Research Unit (PSSRU), University of Kent. 2014. <https://www.pssru.ac.uk/pub/uc/uc2014/full-with-covers.pdf> . Accessed 19 December 2017.
4. Chemist and Druggist. Average locum pay rate across UK increases for first time in a decade. Chemist and Druggist, London. 2017. <https://www.chemistanddruggist.co.uk/news/UK-locum-pay-rate-increases>. Accessed 19 December 2017.
