# Supplementary material for: General practitioner practice-based pharmacist input to medicines optimisation in the UK: pragmatic, multicenter, randomised, controlled trial
Source: J Pharm Policy Pract. 2021 Jan 4;14:4. doi: 10.1186/s40545-020-00279-3 (PMC7784025; doi:10.1186/s40545-020-00279-3)
Supplement: Supplementary file 2 — Additional file 2. Secondary outcome measurement details. [file 40545_2020_279_MOESM2_ESM.docx]

**Additional file 2.** Secondary outcome measurement details

1. Number of medication-related problems [1]

The validated MRP classification system includes six main categories, nine subcategories and 27 assessments. The six categories are indication, effectiveness, safety, knowledge, adherence and miscellaneous. The indication category has two subcategories which are: unnecessary drug therapy and untreated condition, while effectiveness and safety categories have four subcategories: ineffective/incomplete drug therapy, inappropriate dose regimen, actual or potential adverse drug reaction and actual or potential clinically important drug interaction. Knowledge, adherence and miscellaneous categories have no subcategories, and are simply classified as inappropriate knowledge, inappropriate adherence and miscellaneous. This system has shown good reproducibility and inter-rater agreement [1].

1. Medication appropriateness index (MAI) score [2]

The MAI is the most commonly used implicit tool to quantify prescribing appropriateness and changes in quality of prescribing in intervention studies in clinical settings, including primary care [3]. It can be applied to all prescribed medications through scoring of ten explicit weighted criteria: indication, effectiveness, dosage, correct directions, practical directions, drug-drug interactions, drug-disease interactions, duplication, duration of treatment and cost. Indication and effectiveness are triple weighted; dosage, correct directions, drug–drug interactions, or drug–disease interactions are double weighted; and practical directions, duplication, duration of treatment and cost are single weighted. MAI scores per drug range from 0 to 18 where a score of 0 indicates completely appropriate and 18 indicates completely inappropriate prescribing. Summing of MAI scores per drug for the whole regimen received by an individual patient provides an overall MAI score per patient [4].

MAI scoring is a time-consuming process, with approximately 10 minutes required per medication. MAI scoring in the present study was therefore carried out for a random sample of one third of the total sample of patients in each practice, in intervention and control patient groups, at baseline and at the end of the study

[2,3,4,5,6,7].

1. Medication adherence report scale (MARS) [8]

The MARS is a validated questionnaire that assesses self-reported adherence to prescribed medications through five statements. It is convenient to use, has good internal consistency and covers both unintentional (*I forget to take them*) and intentional non-adherence (*I alter the dose, I stop taking them for a while, I decide to miss out a dose, I take less than instructed*). Each statement frequency (*always, often, sometimes, rarely, never*) is selected by the participating patient using a 5-point Likert scale with the range of scores from 1 point for “always” and 5 points for “never”. The total MARS score therefore ranges from 5 to 25. Higher scores represent better adherence levels [8,9]..

1. Beliefs about medicines questionnaire (BMQ) [10]

The BMQ is a validated self-report questionnaire with ten statements that measure the beliefs of patients about their medication. It consists of two five-item subscales that measure the patient’s “necessity beliefs” and their “concerns” about their medication. Participants are required to provide a response to each statement using a 5-point Likert scale ranging from a score of 1 which represents strongly disagree to a score of 5 which represents strongly agree. The total score of the necessity scale and the concern scale are calculated separately with scores ranging from 5 to 25. The differential scores between necessity and concern scales therefore range from -20 to +20. Higher scores for the necessity scale are associated with greater patient perceived need for their medication to control their disease and maintain or improve their health. On the other hand, higher scores for the concern scale are associated with higher patient concerns about harmful or adverse effects that are possible from long-term use of the medicine in question and worries regarding becoming dependent on their medications [10].

1. Health-related quality of life (HR-QOL) (EQ-5D-5L) [11]

The EQ-5D-5L (EuroqQol-5 Dimensions; 5 Levels) is a self-reported questionnaire for use by patients to describe their health perception. It comprises two sections. The first section is descriptive and consists of five dimensions (mobility, self-care, usual activities, pain or discomfort, and anxiety or depression) and each dimension has five levels of severity (level 1: no problems, level 2: slight problems, level 3: moderate problems, level 4: severe problems, and level 5: extreme problems). This approach can define a total of 3125 possible combinations of individual’s health status in terms of a 5-digit code. EQ-5D-5L utility index ranges from the worst (-1) to the best (1) health you can imagine [12,13].

The second section is the EQ visual analogue scale (VAS) where general health state is rated on a 20 cm scale from the worst (0) to the best (100) health you can imagine [12,13]. This tool is also used for clinical and economic appraisal and is a preferred measure in adults for cost-effectiveness analyses where health effects are expressed in QALYs [11,14,15].

1. Patient satisfaction with GP practice-based pharmacist service

The bespoke patient satisfaction questionnaire used in the present study consisted of five items and was designed to measure the satisfaction level of participating intervention group patients. Each statement reflected patient views about the new pharmacy service (*I was happy with the new pharmacy service; I found the information given to me by the pharmacist to be beneficial; I feel that the pharmacist has helped me with my medicines; I feel that the number of pharmacist appointments was enough and I would recommend making this service available for other patients*). The response for each item could be selected using a 5-point Likert scale from strongly agree to strongly disagree. A separate comment space was also provided to allow patients to provide free text feedback (please see below).

## Patient Satisfaction Questionnaire

**Study Title**: GP practice based pharmacist input to medicines optimisation.

**Patient Study Number: Date:**

This survey asks for your views about the new pharmacy service.

Please take the time to read and answer each question carefully. Place an ‘X’ in the box that best represents your response. Please select only one choice for each item*.*

|  | strongly agree | agree | uncertain | disagree | strongly disagree |
| --- | --- | --- | --- | --- | --- |
| 1. I was happy with the new pharmacy service |  |  |  |  |  |
| 1. I found the information given to me by the pharmacist to be beneficial |  |  |  |  |  |
| 1. I feel that the pharmacist has helped me with my medicines |  |  |  |  |  |
| 1. I feel that number of pharmacist appointments was enough. |  |  |  |  |  |
| 1. I would recommend making this service available for other patients. |  |  |  |  |  |

Any other comments:

|  |
| --- |

Thank you for completing.

References

1. AbuRuz SM, Bulatova NR, Yousef AM. Validation of a comprehensive classification tool for treatment related problems. Pharm Word Sci. 2006;28:222–232.
2. Hanlon JT, Schmader KE, Samsa GP, Weinberger M., Uttech KM., Lewis IK., Cohen HJ, Feussner JR. A Method for Assessing Drug-Therapy Appropriateness. J Clin Epidemiol. 1992;45:1045–1051.
3. Hanlon JT, Schmader KE. The Medication Appropriateness Index at 20: Where it Started, Where it has been and Where it May be Going. Drugs Aging. 2013;30:893-900.
4. Samsa GP, Hanlon JT, Schmader KE, Weinberger M, Clipp EC, Uttech KM, Lewis IK, Landsman PB, Cohen HJ. A summated score for the medication appropriateness index: development and assessment of clinimetric properties including content validity. J Clin Epidemiol. 1994;47:891-896.
5. Bregnhoj L, Thirstrup S, Kristensen MB, Sonne J. Reliability of a modified medication appropriateness index in primary care. European Journal of Clinical Pharmacology. 2005;61(10):769-73
6. O'Connor MN, Gallagher P, O'Mahony D. Inappropriate prescribing: criteria, detection and prevention. Drugs Aging. 2012;29:437-52.
7. West LM, Cordina M, Cunningham S. Clinical pharmacist evaluation of medication inappropriateness in the emergency department of a teaching hospital in Malta. Pharmacy Practice. 2012;10:181-187
8. Horne R, Weinman J. Self-regulation and self-management in asthma: exploring the role of illness perceptions and treatment beliefs in explaining non-adherence to preventer medication. Psychology and Health. 2002;14:1–24.
9. Tommelein E, Mehuys E, Van Tongelen I, Brusselle G, Boussery K. Accuracy of the Medication Adherence Report Scale (MARS-5) as a Quantitative Measure of Adherence to Inhalation Medication in Patients With COPD. Ann Pharmacother. 2014;48: 589–595.
10. Horne R, Weinman J, Hankins M. The beliefs about medicines questionnaire: The development and evaluation of a new method for assessing the cognitive representation of medication. Psychology and Health. 1999;14: 1-24.
11. EuroQol Group. EuroQol-a new facility for the measurement of health-related quality of life. Health Policy. 1990;16:199–208.
12. Herdman M, Gudex C, Lloyd A, Janssen M, Parkin D, Bonsel G, Badia X. Development and preliminary testing of the new five-level version of EQ-5D (EQ-5D-5L). Qual life Res. 2011;20:1727-36.
13. Van Reenen M, Janssen B. EQ-5D-5L user guide. Basic information how to use the EQ-5D-5L instrument. 2015. Available from <https://apersu.ca/wp-content/uploads/2016/02/EQ-5D-5L_User-Guide.pdf> (accessed on 25/05/2016).
14. Bullinger M, Quitmann, J. Quality of life as patient-reported outcomes: principles of assessment. Dialogues in Clinical Neuroscience. 2014;16:137–145.
15. National Institute for Health and Clinical Excellence. Guide to the methods of technology appraisal. London: National Institute for Health and Clinical Excellence. 2013. <https://www.nice.org.uk/process/pmg9/resources/guide-to-the-methods-of-technology-appraisal-2013-pdf-2007975843781>. Accessed 25 May 2016.
