# Supplementary material for: General practitioner practice-based pharmacist input to medicines optimisation in the UK: pragmatic, multicenter, randomised, controlled trial
Source: J Pharm Policy Pract. 2021 Jan 4;14:4. doi: 10.1186/s40545-020-00279-3 (PMC7784025; doi:10.1186/s40545-020-00279-3)
Supplement: Supplementary file 3 — Additional file 3. Classes of medications attracting GP practice-based pharmacist interventions at baseline and the third assessment in intervention patients who received three pharmacist interventions. [file 40545_2020_279_MOESM3_ESM.docx]

**Additional file 3.** Classes of medications attracting GP-practice based pharmacist interventions at baseline and the third assessment in intervention patients who received three pharmacist interventions

| **Class of medications attracting interventions at baseline*** | **N** | **%** |
| --- | --- | --- |
| Statins | 34 | 12.3 |
| Proton pump inhibitors | 17 | 6.2 |
| Opioid analgesics | 13 | 4.7 |
| Loop diuretics | 10 | 3.6 |
| Iron | 9 | 3.3 |
| Corticosteroid-Beta2-adrenoceptor agonists combinations | 8 | 2.9 |
| Bulk-forming laxatives | 8 | 2.9 |
| Antiepileptics | 8 | 2.9 |
| Calcium channel blockers | 7 | 2.5 |
| Mucolytics | 7 | 2.5 |
| Xanthine oxidase inhibitors | 7 | 2.5 |
| Angiotensin-converting enzyme inhibitors | 6 | 2.2 |
| Antihistamines | 6 | 2.2 |
| Calcium salts | 6 | 2.2 |
| Vitamin D and analogues | 5 | 1.8 |
| Corticosteroids | 5 | 1.8 |
| Alfa-adrenoceptor blockers | 5 | 1.8 |
| Non-opioid analgesics | 5 | 1.8 |
| Beta-adrenoceptor blockers | 5 | 1.8 |
| Biguanides | 5 | 1.8 |
| Factor XA inhibitors | 5 | 1.8 |
| Antiplatelets | 5 | 1.8 |
| Antidepressant tetracyclics | 4 | 1.4 |
| Angiotensin-II receptor antagonists | 4 | 1.4 |
| Beta2-adrenoceptor agonists | 4 | 1.4 |
| Bisphosphonates | 4 | 1.4 |
| Insulins | 4 | 1.4 |
| Nitrates | 4 | 1.4 |
| Non-steroidal anti-inflammatory drugs | 4 | 1.4 |
| Thyroid hormones | 4 | 1.4 |
| Antimuscarinics | 4 | 1.4 |
| Vitamin K antagonists | 3 | 1.1 |
| Histamine analogues | 3 | 1.1 |
| Thiazide and related diuretics | 3 | 1.1 |
| Stimulant laxatives | 2 | 0.7 |
| Selective serotonin re-uptake inhibitors | 2 | 0.7 |

**Additional file 3.** Classes of medications attracting GP-practice based pharmacist interventions at baseline and the third assessment in intervention patients who received three pharmacist interventions (continued)

| **Class of medications attracting interventions at baseline*** | **N** | **%** |
| --- | --- | --- |
| Second generation antipsychotics | 2 | 0.7 |
| Dipeptidylpeptidase-4 inhibitors | 2 | 0.7 |
| Oestrogens | 2 | 0.7 |
| Cholesterol absorption inhibitors | 2 | 0.7 |
| Potassium | 2 | 0.7 |
| Quinine salts | 2 | 0.7 |
| Sulfonylureas | 2 | 0.7 |
| Hypnotics, sedatives and anxiolytics | 2 | 0.7 |
| Multivitamins | 2 | 0.7 |
| Osmotic laxatives | 1 | 0.4 |
| Selective serotonin receptor agonist | 1 | 0.4 |
| Antiarrhythmic | 1 | 0.4 |
| Tetracyclines | 1 | 0.4 |
| Dopamine receptor antagonist | 1 | 0.4 |
| Serotonin uptake inhibitor | 1 | 0.4 |
| Triazole antifungal | 1 | 0.4 |
| H2-receptor antagonist | 1 | 0.4 |
| Antimuscarinic-Beta2-adrenoceptor agonists combinations | 1 | 0.4 |
| Bile acid sequestrant | 1 | 0.4 |
| Decongestant | 1 | 0.4 |
| Antidepressant tricyclics | 1 | 0.4 |
| Potassium-sparring diuretic | 1 | 0.4 |
| Other lipid modifying drugs | 1 | 0.4 |
| Glucagon-like peptide-1 receptor agonist | 1 | 0.4 |
| Purine synthesis inhibitor | 1 | 0.4 |
| Antimetabolite | 1 | 0.4 |
| Leukotriene receptor antagonist | 1 | 0.4 |
| Artificial saliva product | 1 | 0.4 |
| Sodium Glucose Co-transporter 2 inhibitor | 1 | 0.4 |
| Sodium chloride | 1 | 0.4 |
| Vasodilator | 1 | 0.4 |
| Vitamin B group | 1 | 0.4 |

**Additional file 3** Classes of medications attracting GP-practice based pharmacist interventions at baseline and the third assessment in intervention patients who received three pharmacist interventions (continued)

| **Class of medications attracting interventions at the third assessment*** | **N** | **%** |
| --- | --- | --- |
| Proton pump inhibitors | 9 | 13.6 |
| Statins | 6 | 9.1 |
| Opioid analgesics | 4 | 6.1 |
| Calcium channel blockers | 4 | 6.1 |
| Insulins | 3 | 4.5 |
| Quinine salts | 3 | 4.5 |
| Angiotensin-converting enzyme inhibitors | 2 | 3.0 |
| Alfa-adrenoceptor blockers | 2 | 3.0 |
| Antiepileptics | 2 | 3.0 |
| Biguanides | 2 | 3.0 |
| Corticosteroid-Beta2-adrenoceptor agonists combinations | 2 | 3.0 |
| Corticosteroids | 2 | 3.0 |
| Bulk-forming laxatives | 2 | 3.0 |
| Sulfonylureas | 2 | 3.0 |
| Nitrate | 1 | 1.5 |
| Antiarrhythmic | 1 | 1.5 |
| Antidepressant tricyclic | 1 | 1.5 |
| Antimuscarinic | 1 | 1.5 |
| Antiplatelet | 1 | 1.5 |
| Dipeptidylpeptidase-4 inhibitor | 1 | 1.5 |
| Ocular lubricant | 1 | 1.5 |
| Histamine analogue | 1 | 1.5 |
| Antihistamine | 1 | 1.5 |
| Hypnotics, sedatives and anxiolytic | 1 | 1.5 |
| Iron | 1 | 1.5 |
| Mucolytic | 1 | 1.5 |
| Non-steroidal anti-inflammatory drug | 1 | 1.5 |
| Testosterone | 1 | 1.5 |
| Thiazide and related diuretic | 1 | 1.5 |
| Thyroid hormone | 1 | 1.5 |
| Other antispasmodic | 1 | 1.5 |
| Vitamin B group | 1 | 1.5 |
| Xanthine oxidase inhibitor | 1 | 1.5 |
| Sulfonylurea | 1 | 1.5 |
| *Class of medication according to the relevant chapter of the British National Formulary 76^th^ edition [1]. | | |

Reference

1. British Medical Association and the Royal Pharmaceutical Society of Great Britain. British National Formulary. 76^th^ ed. UK: BMJ Publishing Group. 2018
