# Supplementary figures and images for: General practitioner practice-based pharmacist input to medicines optimisation in the UK: pragmatic, multicenter, randomised, controlled trial
Source: J Pharm Policy Pract. 2021 Jan 4;14:4. doi: 10.1186/s40545-020-00279-3 (PMC7784025; doi:10.1186/s40545-020-00279-3)

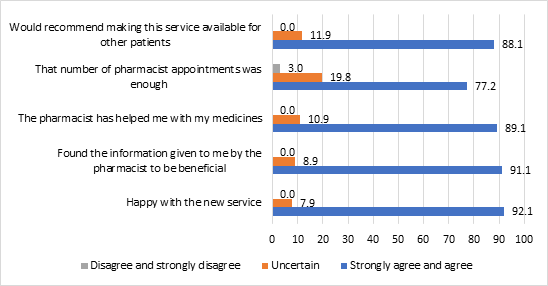

Supplement: Supplementary file 4 — Additional file 4. Patient satisfaction questionnaire results. [file 40545_2020_279_MOESM4_ESM.tif]

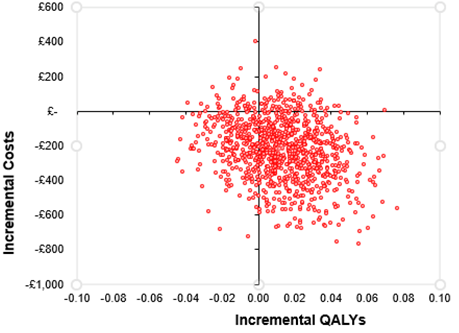

Supplement: Supplementary file 6 — Additional file 6. The cost–effectiveness plane. [file 40545_2020_279_MOESM6_ESM.tif]
