# Supplementary material for: General practitioner practice-based pharmacist input to medicines optimisation in the UK: pragmatic, multicenter, randomised, controlled trial
Source: J Pharm Policy Pract. 2021 Jan 4;14:4. doi: 10.1186/s40545-020-00279-3 (PMC7784025; doi:10.1186/s40545-020-00279-3)
Supplement: Supplementary file 5 — Additional file 5. Cost utility analysis using the mean data over the 6-month study period (per protocol approach). [file 40545_2020_279_MOESM5_ESM.docx]

**Additional file 5**. Cost utility analysis using the mean data over the six-month study period (per protocol approach)

| **Resource use** | **Intervention** | **Control** | **Intervention-control;** | **P value** |
| --- | --- | --- | --- | --- |
|  | **n=113* (48.7%)** | **n=119* (51.3%)** | **mean difference (95%CI)** |  |
| Total cost of Healthcare resource utilisation^a^, mean ± SD | 779.5 ± 1151.6 | 1039.1 ± 1562.8 | -259.5(-624.8, 98.1) | 0.113^b^ |
| Cost of pharmacist intervention,  mean ± SD | 30.5 ± 2.1 | 0 |  |  |
| **Overall cost** (healthcare and intervention), mean ± SD | 810.1 ± 1151.8 | 1039.1 ± 1562.8 | -229.0 (-594.6, 128.2) | 0.289^b^ |
| **EQ-5D-5L utility scores**, mean ± SD | 0.554 ± 0.321 | 0.506 ± 0.323 | 0.047 (-0.042, 0.129) | 0.175^b^ |
| **QALY** in 0.5 years, mean ± SD | 0.277 ± 0.161 | 0.253 ± 0.162 | 0.024 (-0.021, 0.065) | 0.175^b^ |
| **Incremental cost/incremental QALY (ICER)**  **-£229/0.024 QALYs = Dominant** | | | | |
| ^*^Five patients’ healthcare resource utilisation data were not available and four patients had 20 or more days of length of stay at baseline or six-month follow-up, eight patients had missing EQ5D5L utility scores.  ^a^All healthcare resource utilisation without intervention cost.  ^b^ Mann Whitney U test (between control vs intervention) | | | | |
