# Supplementary material for: General practitioner practice-based pharmacist input to medicines optimisation in the UK: pragmatic, multicenter, randomised, controlled trial
Source: J Pharm Policy Pract. 2021 Jan 4;14:4. doi: 10.1186/s40545-020-00279-3 (PMC7784025; doi:10.1186/s40545-020-00279-3)
Supplement: Supplementary file 7 — Additional file 7. Sensitivity analysis associated with the cost utility analysis. [file 40545_2020_279_MOESM7_ESM.docx]

**Additional file 7**. Sensitivity analysis associated with the cost-utility analysis

| **Sensitivity analysis scenarios** | **Intervention**  **n=113**  **(48.7%)** | **Control**  **n=119**  **(51.3%)** | **Intervention-control; mean difference (95%CI)** | **ICER (£/QALY)** |
| --- | --- | --- | --- | --- |
| 50% increase in total healthcare cost,  Mean overall cost ± SD | 1199.8 ± 1727.7 | 1039.1 ± 1562.8 | 160.8 (-265.6, 614.4) | 6700 |
| 50% increase in cost of intervention  Mean overall cost ± SD | 825.3 ± 1152.0 | 1039.1 ± 1562.8 | -213.7 (-579.6, 143.3) | Dominant |
| 50% decrease in QALYs gained (0.012)  Mean overall cost ± SD | 810.1 ± 1151.8 | 1039.1 ± 1562.8 | -229.0 (-594.6, 128.2) | Dominant |
